# Native microorganisms as natural enhancers in craft beer and wine production

**DOI:** 10.3389/fmicb.2026.1754472

**Published:** 2026-03-10

**Authors:** Carlos Caiza-Valencia, Andrés Izquierdo Romero, Eliana Veloz-Villavicencio, Jonathan Coronel-León

**Affiliations:** 1 Facultad de Ingeniería en Mecánica y Ciencias de la Producción (FIMCP), Escuela Superior Politécnica del Litoral (ESPOL), Guayaquil, Ecuador; 2 Center for Nanoscience and Nanotechnology (CENCINAT), Universidad de las Fuerzas Armadas ESPE, Sangolquí, Ecuador; 3 Department of Life and Agricultural Sciences, Universidad de las Fuerzas Armadas ESPE, Sangolquí, Ecuador; 4 Institute of Agrifood Research and Technology (IRTA), Food Safety and Functionality Programme, Monells, Spain; 5 Centro de Investigaciones Biotecnológicas del Ecuador (CIBE), Escuela Superior Politécnica del Litoral (ESPOL), Guayaquil, Ecuador

**Keywords:** native microorganisms, fermentation, non-*Saccharomyces* yeast, lactic acid bacteria, sustainability

## Abstract

The use of native microorganisms in craft beer and wine production represents a transformative approach that significantly enhances quality, sensorial complexity, and authenticity of the beverages. Integrating these microorganisms facilitates terroir expression, reduces dependence on commercial monocultures, and promotes sustainability by preserving native microbial biodiversity, which is vital to the identity of fermented craft beverages. This review analyzes the main roles of native yeasts, including *Saccharomyces cerevisiae, Brettanomyces bruxellensis, Lachancea thermotolerans, Torulaspora delbrueckii, Pichia kudriavzevii*, and *Hanseniaspora* spp., in shaping the biochemical and sensory profiles of craft beer and wine. It also provides a summary of the functions of lactic acid bacteria (LAB; *Lactobacillus, Leuconostoc, Pediococcus, Oenococcus*) in malolactic fermentation and flavor development, as well as the effects of acetic acid bacteria (AAB; *Acetobacter, Gluconobacter*) on oxidative complexity in select beverage styles. This work also highlights the use of mixed inoculation strategies and targeted strain selection to optimize fermentation kinetics, stress tolerance, and enzymatic activities, particularly those that enhance the liberation of aroma precursors. Additionally, challenges such as fermentation consistency, microbiological safety, and the need for standardized processes are also discussed. This framework provides essential insights to researchers and craft producers seeking innovation, product differentiation, and cultural preservation in the craft brewing and winemaking sectors, supporting regional economies and global biodiversity conservation through the scientifically grounded exploitation of native microorganisms.

## 1 Introduction

An emerging challenge in craft beer and wine production is the marked decline in microbial diversity used as starter cultures, leading to reduced product uniqueness and sensory complexity. This loss is primarily a consequence of industrial-scale processes that prioritize efficiency, predictability, and standardization, often replacing regionally adapted, naturally diverse microbial consortia with monoculture fermentations (Burini, 2022; Grijalva-Vallejos et al., 2021; Siqueira, 2023). The adoption of pure-culture fermentations, pioneered in the late nineteenth century with Emil Christian Hansen's isolation of *Saccharomyces carlsbergensis* (now *Saccharomyces pastorianus*), while affording substantial reproducibility and reliability, has inadvertently marginalized traditional mixed and spontaneous fermentations (Basile et al., 2025; Gutiérrez et al., 2018; Postigo et al., 2023b). As a consequence, the reduction of product variety in beer and wine markets is linked to the limited genetic and metabolic range of commercial starter cultures, which lack the dynamic interactions among native yeast, bacteria, and environmental factors traditionally responsible for conferring unique qualities to craft beverages (Grijalva-Vallejos et al., 2020; Liu, 2020). On the other hand, consumers' preferences change over time, increasing the interest in beverages that represent regional identity, sensory distinctiveness, and cultural authenticity (Burini, 2022; Grijalva-Vallejos et al., 2021). Therefore, to address this significant constraint, the resurgence of native microorganism use, which contributes positively to fermentation and aroma formation in brewing and winemaking, such as non-*Saccharomyces* species, also called non-conventional yeasts, whether in pure or mixed-culture starters, is being advocated as an innovative strategy to renew, enhance, and diversify microbial resources in fermentation processes. For instance, in mixed-culture fermentations, the order of inoculation of native *Saccharomyces cerevisiae* and non-conventional yeasts critically affects fermentation kinetics and the final sensory profile. It should therefore be adapted to the targeted beer or wine style rather than treated as a fixed parameter (Zavaleta et al., 2025).

Modern craft producers are progressively adopting spontaneous fermentations or cultivating the native starters sourced from their own environments, reclaiming the concept of microbial terroir and leveraging a vast and underutilized reservoir of metabolic functions (Grijalva-Vallejos et al., 2021; Gutiérrez et al., 2018; Sannino et al., 2019; Steensels and Verstrepen, 2014; van Rijswijck, 2017).

Native microbial communities, including non-*Saccharomyces* yeasts such as *Brettanomyces bruxellensis, Lachancea thermotolerans, Torulaspora delbrueckii, Pichia kudriavzevii*, and *Hanseniaspora* spp., have been reported to have significant metabolic ways that enrich flavor profiles, textures, and aromatic compounds through the production of diverse metabolites, often exceeding the sensory complexity achievable by *S. cerevisiae* monocultures (Gamero et al., 2020; Nasuti et al., 2023; van Rijswijck, 2017). In addition, LAB such as *Lactobacillus, Leuconostoc, Pediococcus*, and *Oenococcus* contribute through malolactic fermentation, acid modulation, and enhanced microbial stability. In contrast, the controlled integration of acetic acid bacteria (AAB), such as *Acetobacter* and *Gluconobacter*, introduces oxidative complexity to select beer styles or barrel-aged wines (De Roos et al., 2018; Mora-Villalobos et al., 2020; Ponomarova et al., 2017).

**Figure 1:**
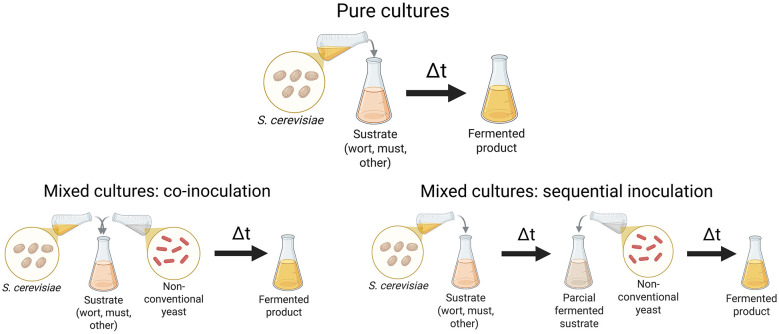
Graphical summary of pure cultures and mixed cultures (sequential inoculation and co-inoculation). The scheme illustrates only one possible inoculation sequence and is intended as a conceptual example; in practice, different orders and timings of yeast addition can be applied, and no single configuration is universally optimal across fermentation processes. Created with BioRender.com.

Recent advancements in high-throughput sequencing, metagenomics, and metabolomics have enabled the characterization and propagation of native microbial communities, enabling controlled applications that preserve microbial terroir while enhancing process reliability (Beltrán, 2021; Bintsis, 2018; Mendoza et al., 2017; Vallejo et al., 2013). Mixed and sequential inoculation strategies (Figure 1), co-fermenting *Saccharomyces* with non-*Saccharomyces* yeasts or LAB, embody a synthesis of tradition and biotechnological innovation, optimizing fermentation kinetics, mitigating spoilage, and enriching functional beverage qualities (Capozzi et al., 2019; Dallos et al., 2024; Jussier et al., 2006).

Nevertheless, the strategic implementation of native microorganisms presents operational challenges: spontaneous and mixed fermentations can be variable and require robust protocols to ensure consistency, control, and food safety (Alencar and Hikichi, 2019). Addressing these challenges requires precise microbial selection, a deeper understanding of microbial ecology, and a strict process of standardization (Alencar and Hikichi, 2019; Capozzi et al., 2019; Dysvik et al., 2020b). Thus, the renewed focus on native microorganisms, pure or mixed cultures, represents an opportunity to improve small-batch and industrial fermentations and a forward-looking strategy to unlock the sensory, functional, and cultural potential of craft beer and wine (Alencar and Hikichi, 2019; Bader et al., 2019; Capozzi et al., 2015; Shruthi et al., 2024). Based on the aforementioned, the present work analyzes the main roles of native yeasts and LAB in shaping the biochemical and sensory profiles of craft beer and wine. Also, provide an overview of the use of mixed inoculation strategies and targeted strain selection to optimize fermentation kinetics, stress tolerance, and enzymatic activities. It discusses the importance of native microorganisms in sustainability, biodiversity conservation, and their potential for biotechnological innovation. Finally, challenges such as fermentation consistency, microbiological safety, and the need for standardized processes in craft breweries and wineries were analyzed.

## 2 Microbial diversity in brewing and winemaking

The development of novel fermented beverages with distinctive sensory and functional attributes requires, as a first step, a thorough understanding of the microbial diversity associated with specific environments destined for fermentation. Characterizing these microbial communities is essential for elucidating their potential roles in shaping metabolic pathways, influencing fermentation dynamics, and ultimately determining the organoleptic and qualitative properties of the final product. In this context, microbial diversity plays a fundamental role in driving the complex biochemical and sensory transformations that characterize beer and wine fermentation (Boulton, 2019). This process involves a diverse array of microorganisms, primarily yeasts and LAB, each contributing to distinct metabolic activities (Bamforth, 2005; Boulton, 2019). For this reason, this section provides useful information about microbial diversity and their metabolic ways related to the production of desirable characteristics that can improve the quality of craft beer and wine.

Alcoholic fermentation drives wine and beer production. Yeast and LAB enzymes convert fermentable sugars, such as glucose, maltose, maltotriose, and fructose, present in grape must or wort into ethanol and carbon dioxide (Grumezescu and Holban, 2019). At the same time, many secondary metabolites, such as esters, higher alcohols, aldehydes, volatile acids, glycerol, and diacetyl, are produced; these molecules define aroma, taste, and mouthfeel (Bader et al., 2019; Cheng S. et al., 2025). Fermentation can rely on a single strain or a mixed community; species succession determines sugar uptake and metabolite output. The result guides beverage style and enables brewers to craft new, innovative and functional products (Bader et al., 2019; Cheng S. et al., 2025).

The exploitation of non-*Saccharomyces* yeasts and bacteria isolated from spontaneous fermentations (see Table 1) is a notable advancement in craft beer and wine production (Avedovech, 1988; Dysvik, 2019; Esteve-Zarzoso et al., 1998). These microorganisms secrete key enzymes, such as β-glucosidase, protease, and β-lyase, which catalyze the release of otherwise bound aroma precursors, substantially enhancing sensory complexity and depth beyond the capacity of traditional *Saccharomyces cerevisiae* strains. The enzymatic liberation of these aromatic compounds imparts unique flavor profiles and broadens the organoleptic spectrum, meeting the evolving preferences of consumers seeking differentiated craft products (Esteve-Zarzoso et al., 1998; Qin et al., 2025).

**Table 1:** Microorganisms involved in brewing and winemaking.

**Microorganism**	**Primary role in winemaking/brewing**	**Advantages**	**Disadvntages**	**Impact on sensory profile of the beverage**	**References**
**Yeasts**	
*Saccharomyces cerevisiae*	Convert glucose and fructose into ethanol and carbon dioxide. Produces organic acids and higher alcohols, esters, terpenes and phenolic compounds.	Tolerant to high ethanol level, acidic pH, nutrient limitation, and temperature fluctuation. Proficient ethanol producer due to preference of fermentative metabolism.	May produce undesirable amounts of acetic acid in wine production. Higher alcohols production should be carefully controlled to avoid imbalanced flavors.	Fruity, floral aromas in both wine and beer, refreshing acidity, impact on body and sensory freshness in wine.	Que et al., 2024
*Saccharomyces pastorianus*	Convert primary sugars (maltose, maltotriose, etc.) into ethanol and carbon dioxide. Synthesize higher alcohols, esters and volatile compounds.	Tolerant to escalating ethanol concentrations, low oxygen availability, and cold stress (optimal fermentation temperatures from 7°C to 15°C).	Present limited genetic diversity, which may restrict innovation of new beer styles and flavors.	Clean and less complex sensory profiles compared to *S. cerevisiae*.	Gorter De Vries et al., 2019; Ocvirk et al., 2021
*Saccharomyces kudriavzevii*	Play a role in spontaneous fermentation of Belgian beers. Ferment glucose, sucrose, and maltose.	Cryotolerant, thus contributing metabolically during low-temperature fermentation and extended maturation phases. Tolerate high ethanol concentrations.	Slow fermenter in wine and produces less ethanol when grape juice is used as substrate. In both beer and wine production, should be used in tandem with other *Saccharomyces* species to obtain a improved fermentation outcomes.	Intensifies aroma, contributes to fruity and floral notes, and also spicy, smoky and barnyard-like aromas. Influences mouthfeel and caloric content by partial sugar consumption.	De Roos et al., 2018
*Brettanomyces spp*.	Present in traditional spontaneous fermentations in Belgian beers (Lambic and Gueze) and some styles such as Red and White wines.	Metabolically versatile, capable of fermenting complex carbohydrates, thus producing beer with lower caloric content. Produce volatile phenols. Tolerant to high ethanol concentrations, low pH, nutrient depletion, and presence of antimicrobial compounds in beer.	Slower fermenter compared to *S. cerevisiae*. In winemaking, it is considered as a spoilage organism due to the high production of volatile phenols.	Characteristic volatile phenols (“Brett character”), deep aromatic complexity, especially in beers.	Basso et al., 2016; Belda et al., 2017; Berbegal et al., 2017; Qin et al., 2025
*Hanseniaspora spp*.	Prevalent in wine making and, to a lesser extent, in craft beer production. Synthesize esters, higher alcohols and volatile organic compounds (VOCs).	Tolerant to high sugar concentrations and pH variations.	Some species are low resistant to ethanol. Excessive growth might lead to undesirable odors, related to a high content of ethyl acetate.	Mixed fermentations with *S. cerevisiae* enhance complexity, providing fruity, floral and spicy notes in wine and beer production.	García et al., 2016; Zheng et al., 2025
*Metschnikowia pulcherrima*	Predominant during the first stages of fermentation. Produces aromatic esters, alcohols and other VOCs	Elevate ester concentration by showing β-glucosidases, cysteine β-lyases, and amylase activities that liberate bound aroma precursors. Reduce ethanol content and acetic acid concentrations compared to pure cultures of *S. cerevisiae*.	Presents limited fermentative capacity compared to *S. cerevisiae* due to its sensitivity to ethanol concentrations above 4–5% v/v.	Floral and fruity notes, complex and balanced aromas in wines when co-inoculated with *S. cerevisiae*. Improved mouthfeel and freshness.	García et al., 2016; Karaulli et al., 2024
*Torulaspora delbrueckii*	Produce phenolic and aromatic compounds in wine and craft beer fermentation. It has a lower capacity to produce acetic acid, and greater potential for malolactic fermentation.	Exhibit β-lyase and β-glucosidase activities that increase free volatile aroma compounds during wine maturation.	Lower fermentation potential and a lower growth rate than *S. cerevisiae*, so it should be used at an adequate stage of fermentation, in combination with other strains.	Increases fruity/floral aromas in wines and overall flavor complexity, reduces higher alcohol concentration. In beer, provides distinctive aromatic profile in mixed fermentations with *S. cerevisiae*.	Canonico et al., 2020; Maicas and Mateo, 2023; Qin et al., 2025; Tataridis et al., 2013
*Wickerhamomyces anomalus*	Used in mixed and sequential fermentation alongside *Saccharomyces* strains. Produce esters and volatile phenols.	Utilize diverse sugars, including hexoses, pentoses, disaccharides, and polysaccharides. Grow in the absence of oxygen, a wide temperature range, and extreme pH.	Show limited ethanol tolerance compared to dominant fermentative yeasts.	Contribute fruity, floral and spicy notes to beers and wines, improve sensory bouquet, support dominant yeast by enhancing aroma	Basso et al., 2016; Holt et al., 2018; Ravasio et al., 2018
*Pichia spp*.	Present at mid to late stages of fermentation and maturation of wines and beers. Exhibit enzymatic activities that influence aroma development. They have moderate fermentative capacity.	Tolerant to high ethanol concentrations and low pH. Some species are metabolically versatile, allowing diverse sugar consumption. Exhibit probiotic properties.	These species are infrequent in grapes, and there are no adequate selection methods, which result in poor development of commercial strains.	Enhance aromatic profiles in wines.	Huang et al., 2024; Maicas and Mateo, 2023; Mazzucco et al., 2025
*Zygosaccharomyces kombuchaensis*	It has a primary role in mixed wine fermentations. Produces VOCs, and mild acid transformation.	Improve the availability of nitrogenous compounds for supporting other microorganisms during fermentation.	Lower ethanol tolerance compared to *S. cerevisiae*.	Increase flavor intensity and fruity attributes. Enhances aroma and improve texture.	Maicas and Mateo, 2023; Varela, 2016
*Lachancea thermotolerans*	Reduce pH and produce lactic acid, higher alcohols, esters, aldehydes and fatty acids.	Improve microbial stability when used in co-cultures with *S. cerevisiae*. Versatile and safe to increase wine and beer diversity. Shorten fermentation process.	Moderate fermentation power. Therefore, it should be used in mixed fermentation for complete fermentation of must's sugars.	Improve flavor balance and enhance color stability in wines. Modulate acidity in sour beer production, boost flavor intensity, freshness, and mouthfeel. Contribute with floral, honey, and sweet aromas in beer.	Maicas and Mateo, 2023; Postigo et al., 2023a; Zavaleta et al., 2025
*Lactobacillus spp*.	Present in viticultural and brewery niches. Produce lactic and other organic acids, higher alcohols, esters, and phenolic compounds.	Resistant to hop compounds, low pH and high concentrations of ethanol.	Require careful management to avoid undesirable metabolites that affect aroma. Main spoilage agent in beers.	Improve freshness, sourness, and aroma complexity.	Modzelewska et al., 2023
*Pediococcus spp*.	Play a critical role in acidification and sensory development of traditional Lambic beers and specific wines. Important for malolactic fermentation.	Adapt to diverse brewery environments, including oxidative stress and the presence of antimicrobial compounds. Support bacterial growth under anaerobic conditions.	Potential to produce off-flavors if uncontrolled, as well as increased turbidity and viscosity. Some species can produce biogenic amines, considered unsafe due to vasoactive and toxicological properties.	Improve mouthfeel and texture, modulates acidity.	Xu et al., 2020
*Oenococcus oeni*	Primary driver of malolactic fermentation in winemaking. Exhibits notable β-glucosidase activity that releases aroma precursors.	Possess remarkable adaptability to harsh physicochemical conditions (low pH, high ethanol concentration, sulfur dioxide stress) and can reprogram its metabolic pathways to enhance its survival in wine.	It is sensitive to high concentrations of ethanol over 15% (v/v), low temperatures below 18 °C. In wines, it could produce unwanted amounts of acetic acid if uncontrolled.	Acidity balance, smoothness, liberation of glycoside-bound aroma. Due to its contribution to overall microbial stability in wines, it increases the complexity of aroma and flavor.	Grandvalet, 2017; Liu et al., 2025; Vicente et al., 2022
*Acetobacter spp*.	Important during the maturation phase of Lambic beers and aged wines. Converts ethanol to acetic acid, which later decomposes into water and carbon dioxide.	Better adapted to the higher ethanol concentrations, greater ability to metabolize organic acids than other LAB strains.	It can cause spoilage in winemaking when uncontrolled, due to production of vinegar-like sourness.	Provide acidity, aroma depth with fruity, buttery notes.	Bartowsky and Henschke, 2008
*Gluconobacter oxydans*	Shares niche with *Acetobacter* species during the late fermentation and maturation stages in brewing and winemaking.	It has a robust oxidative enzymatic system to convert ethanol into acetic acid, and also potential to inhibit less acid-tolerant spoilage microorganisms.	It is regarded as a spoilage microorganism in both beer and wine if uncontrolled, due to production of excessive acetic acid. Shows less potential to metabolize organic acids compared to *Acetobacter* species.	Contribute with vinegar notes, produces gluconic and ketogluconic acids that strongly bind to SO_2_ and act as antioxidant and antimicrobial in wines.	Bartowsky and Henschke, 2008; Neffe-Skocińska et al., 2022
*Leuconostoc spp*.	Present in brewing and winemaking ecosystems. Ferment sugars into lactic acid.	Potential to inhibit less acid-tolerant spoilage microorganisms.	Compromise sensory quality and product stability if grown excessively. Thus, it requires active monitoring.	Contribute to organoleptic properties due to the production of acetaldehyde, diacetyl and acetoin.	Ruiz et al., 2018

Building on the benefits of individual yeast species, contemporary brewing and winemaking incorporate co-inoculation and sequential fermentation strategies involving non-*Saccharomyces* yeasts, *S. cerevisiae*, and LAB. These methodologies together expand the diversity of flavor compounds (Capozzi et al., 2015; Holt et al., 2018), enhance fermentation robustness by mitigating stuck fermentations and off-flavors, and ensure product consistency and quality. Mastery over microbial interactions during fermentation empowers producers to craft distinctive beverages while simultaneously promoting sensory innovation and opening new market niches (Cheng Z. et al., 2025; Dysvik et al., 2020a).

**Figure 2:**
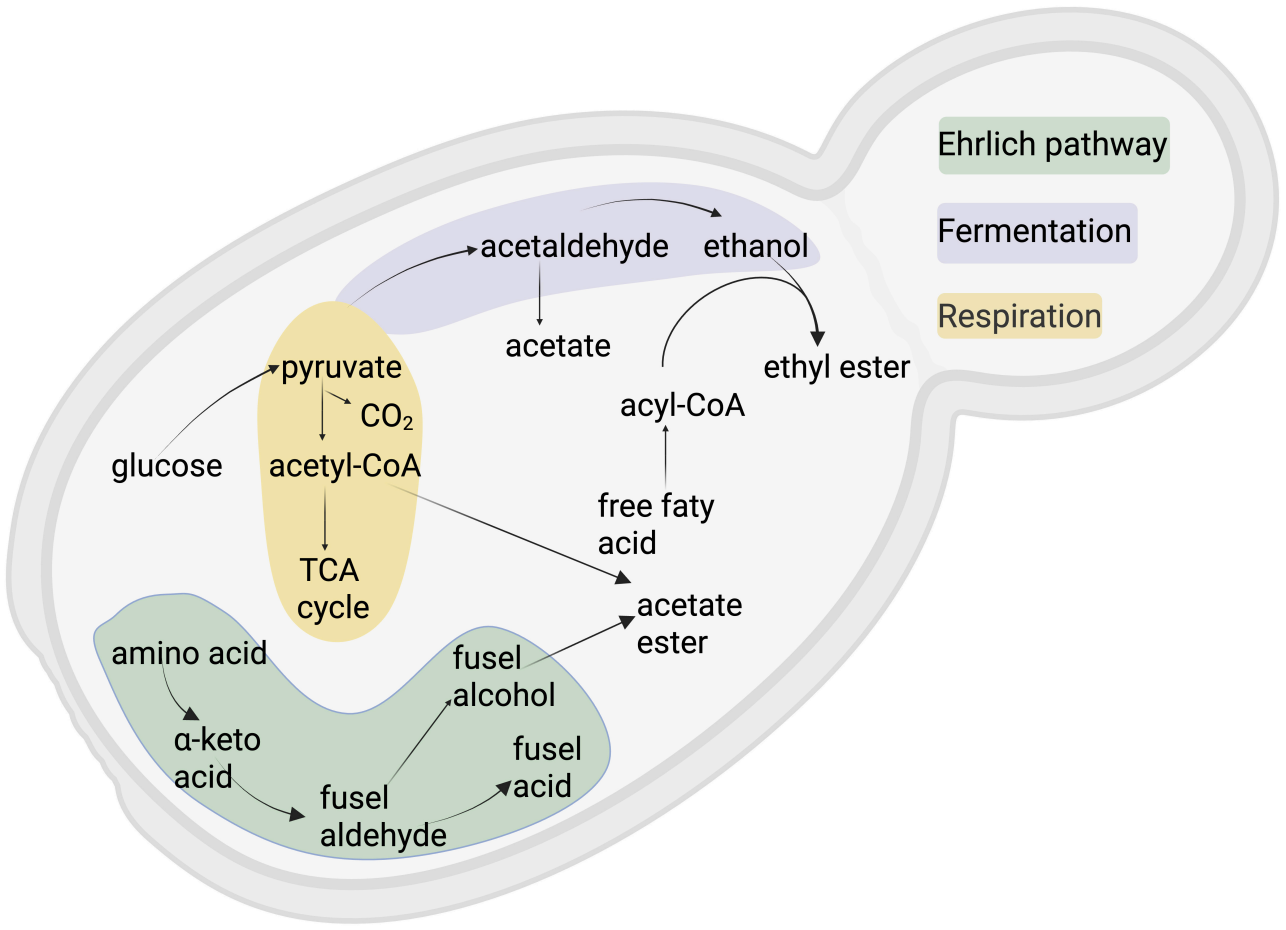
Aroma production mechanism in yeast metabolism. Created with BioRender.com.

Several native yeast species, including *Torulaspora delbrueckii, Hanseniaspora uvarum, Lachancea thermotolerans* and *Metschnikowia pulcherrima*, have been selectively harnessed for their distinctive roles within fermentation ecosystems. Their metabolic activities include the synthesis of fruity esters responsible for tropical and stone fruit aromas, as well as the reduction of volatile acidity (Figure 2), thereby preserving product quality (Bader et al., 2019; Dysvik, 2019; Swiegers et al., 2005). Additionally, these yeasts enhance mouthfeel through elevated glycerol production, a metabolite implicated in improved sensory and health attributes, ultimately enriching the taste and texture of craft beer and wine. (Wedral et al. 2010) describe the presence of *Brettanomyces bruxellensis* phenolic metabolites, notably the volatile phenols 4-ethylphenol and 4-ethylguaiacol, in red wines made from *Vitis vinifera*. At high concentrations, these compounds impart unpleasant phenolic, leathery, smoky, or medicinal notes; however, at low levels, they can contribute positively to aromatic complexity. Similar effects of *B. bruxellensis-*derived volatile phenols have also been reported in white wines by (Nikfardjam et al. 2009). The multifunctionality of these species features the critical importance of targeted strain selection for optimizing both sensory profiles and functional attributes in fermented beverages (Alencar and Hikichi, 2019; Peyer, 2017; Praveen and Brogi, 2025).

Complementing yeast metabolism, LAB from the *Lactobacillus, Pediococcus*, and *Oenococcus* genera have transcended their classical functions in malolactic fermentation. They enhance acid regulation and contribute to aroma complexity by producing volatile compounds that enrich the flavors of sour beers and wines, especially in barrel-aged products (Mora-Villalobos et al., 2020). In wines, these effects are not limited to barrels but also occur during malolactic fermentation in tanks for stabilization, without compromising microbial stability or final product integrity (Avedovech, 1988). Their use in fermentation systems supports the development of novel products while ensuring stable and reproducible performance (Altieri et al., 2017).

## 3 Craft beer and wine in the market: product development and trends

The craft brewing and winemaking industries have undergone remarkable growth and diversification, due to new market trends, technological advancements, and changing consumer preferences. This expansion reflects a global shift toward small-batch products that prioritize authenticity, regional terroir, and sensory uniqueness, driven by the increasing demand for novel flavors, diverse styles, and innovative production methods; for example, in Brazil, native yeast starters have been applied in both the first and second fermentations of sparkling wines produced from unconventional grape varieties such as Chenin Blanc, yielding wines with elevated levels of 2,3-butanediol, 3-ethoxypropan-1-ol, diethyl succinate, and ethyl decanoate, which collectively contribute to an intense fruity aroma profile (Burini, 2022; Raymond Eder and Rosa, 2021). Craft producers can use local raw materials and heritage microbial strains to reinforce the intrinsic connection between product identity and *geographical origin*, thus tightly integrating culture and place into the beverage experience (Alencar and Hikichi, 2019; Gutiérrez et al., 2018).

A promising innovation strategy is to design spontaneous fermentations under controlled conditions by using native non-*Saccharomyces* yeasts and LAB as defined starter cultures (Habschied et al., 2020; Praveen and Brogi, 2025). With technological advances, these kinds of microorganisms are now recognized for their metabolic pathways (Alencar and Hikichi, 2019; Paramithiotis et al., 2024). The impact of non-conventional yeasts and LAB are related to the ability to modulate fermentation, regulate acid production, and enhance aroma synthesis, thereby setting new standards for product differentiation and sensory innovation (Faria-Oliveira et al., 2015; Gschaedler, 2017; Parreira, 2018).

Consumer-driven demand for functional, health-conscious beverages further shapes product development strategies in both sectors (Liu, 2020; Shruthi et al., 2024). Modern consumers seek drinks that integrate wellness benefits with sensory satisfaction, promoting the consumption of low- and non-alcoholic beers and wines, gluten-free and alternative grain-based formulations, and fruit-flavored wine and beer variants (Paramithiotis et al., 2024; Shruthi et al., 2024). These developments align with heightened environmental awareness and global health trends, motivating producers to innovate inclusively and nutritionally (Kozłowski et al., 2021). The advent of gluten-free beers brewed from cereals such as sorghum, millet, and rice, alongside hybrid fruit wines tailored to diverse palates, exemplifies a commitment to expanded accessibility and enhanced nutritional profiles (Marsh et al., 2014).

**Figure 3:**
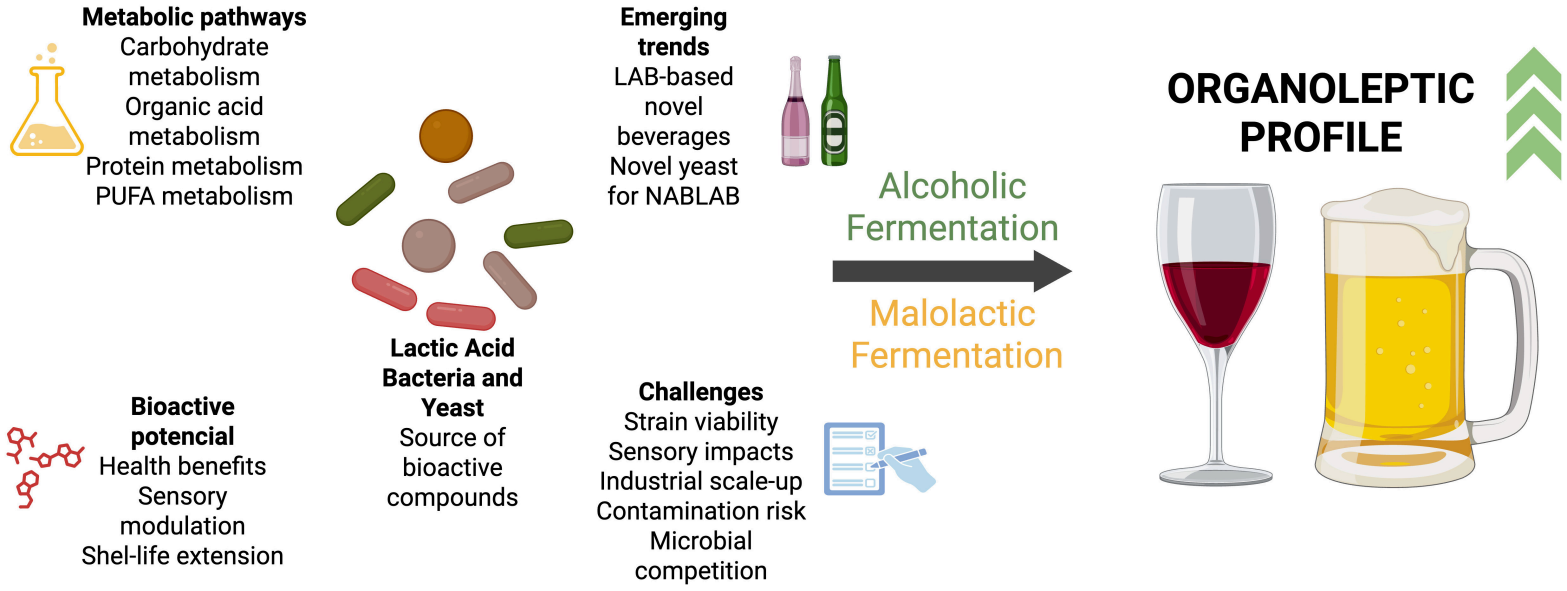
The present and potential future for Yeast and LAB in brewing and winemaking. Created with BioRender.com.

From a technological perspective, leveraging the bio-flavoring capacity of non-*Saccharomyces* yeasts and LAB constitutes a critical frontier for innovation (Figure 3). These microorganisms produce enzymes, such as β-glucosidases, proteases, and β-lyases, that liberate and transform varietal aroma precursors from glycosidic conjugates in wort and must, generating complex bouquets rich in esters, higher alcohols, and phenolic compounds (Kozłowski et al., 2021; Marsh et al., 2014).

Craft breweries and wineries increasingly embrace native yeasts and local ingredients, reinforcing terroir expression as a main factor in product identity and consumer appeal. This strategy not only promotes environmental sustainability through microbial biodiversity conservation but also drives authentic flavor development, which is intimately linked to regional cultural heritage (Capozzi et al., 2015; Paramithiotis et al., 2024). In addition, scientific efforts are increasingly focused on functional and specialty beverage categories, emphasizing their antioxidative properties, probiotic potential, and reduced alcohol content (Burini, 2022; Grijalva-Vallejos et al., 2021).

## 4 Biotechnological innovations and process optimization in brewing and winemaking

Recent advances in strain selection, supported by improved proteomic and metabolomic characterization, have enabled the design of tailored native microbial consortia that could optimize fermentation kinetics (Ellis et al., 2022; Zavaleta et al., 2025). These selection strategies increasingly target specific enzymatic activities, such as glycosidases, esterases, and decarboxylases, which modulate key metabolic pathways governing aroma-precursor release, ester formation, and the balance between acidity and sweetness in the final product. Taken together, this level of precision in microbial and enzymatic management is critical for achieving industrial scalability and reproducible product quality in modern fermentation processes (Parreira, 2018; Zheng et al., 2025).

Biotechnological innovation has also contributed to the emergence of the low- and non-alcoholic beverage market by capitalizing on the unique metabolic pathways of non-*Saccharomyces* yeasts combined with selected LAB (Kozłowski et al., 2021; Vaštík et al., 2025). Some strains of these microorganisms have been reported to produce lower ethanol concentrations while preserving or enhancing desirable sensory attributes, such as acidity, aromatic complexity, and mouthfeel. For instance, the maltose-negative yeast *Saccharomycodes ludwigii* has been successfully employed by (De Francesco et al. 2015) to produce low-alcohol beers. Along the same lines, (Bellut et al. 2020) showed that maltotriose-negative *Lachancea fermentati* strains, isolated from kombucha, can produce low-alcohol beers with lactic acid production, which helps counterbalance residual wort sweetness. In parallel, selected lactic acid bacteria, such as *Lactiplantibacillus plantarum*, have been incorporated into co-fermentation with non-*Saccharomyces* yeasts to reduce alcohol while further shaping the sensory profile (Nyhan et al., 2023). The targeted application of these microbial metabolisms empowers producers to craft beverages that comply with new alcohol regulations without compromising flavor and texture, aligning with evolving consumer demands for healthier options (Kozłowski et al., 2021; Peyer, 2017).

Process optimization includes the design of native microbial starter cultures sourced directly from vineyard and brewery environments (Bozoudi and Tsaltas, 2016; Nasuti et al., 2023). This strategy reinforces terroir expression and regional identity while supporting sustainability by conserving and using local microbial biodiversity. Employing native strains sustains natural microbial ecosystems, yields beverages with distinctive organoleptic profiles that reflect their geographic origin and aligns with broader industrial trends that prioritize ecological stewardship and authenticity in fermentation (Basile et al., 2025).

**Figure 4:**
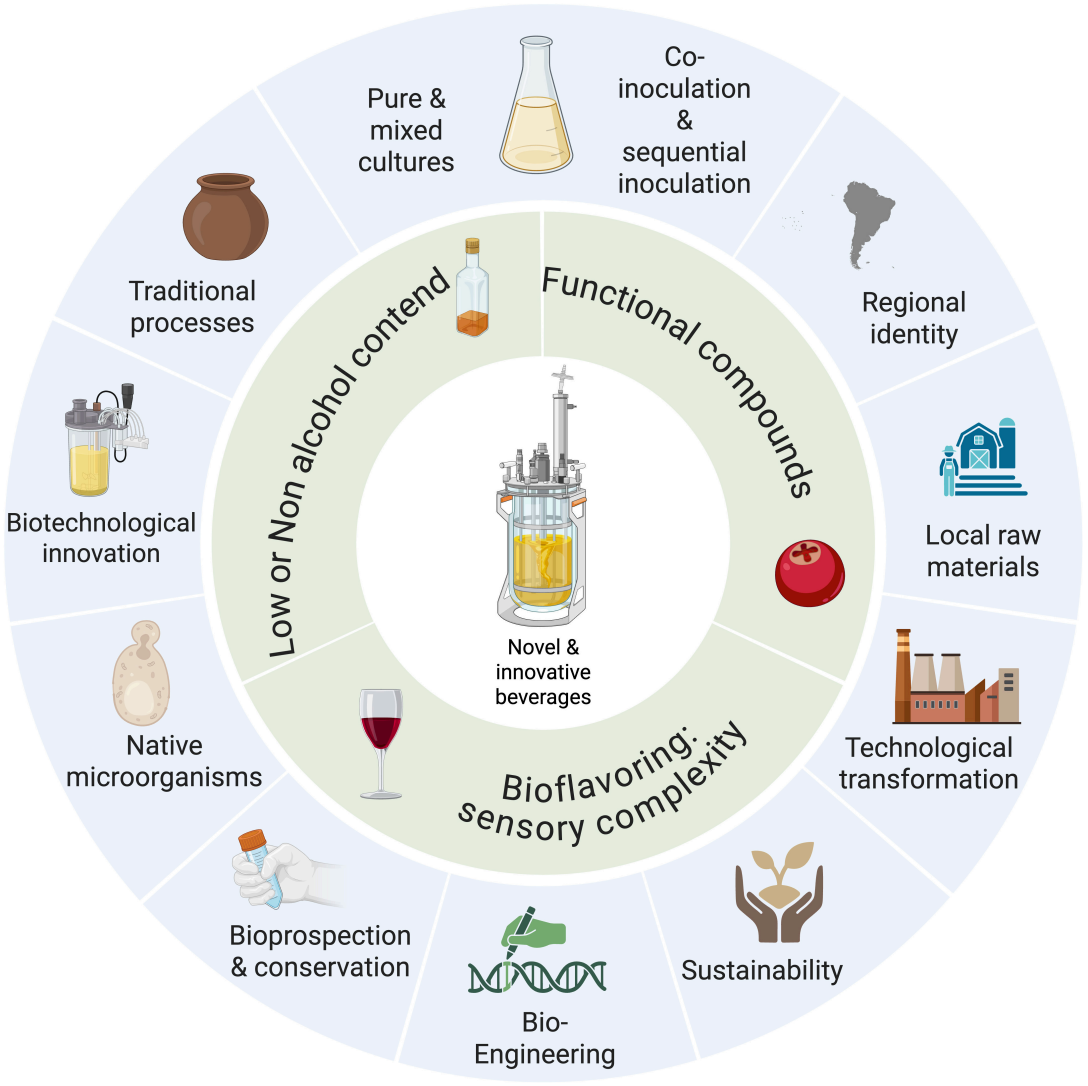
Perspectives in novel functional beverage development. Created with BioRender.com.

The integration of native microbial diversity into controlled fermentation frameworks achieves a balance between process standardization and the development of functional beverages (Figure 4). This dynamic supports the production of probiotic-enriched beers and bioflavored wines with elevated antioxidant levels while concurrently reducing reliance on chemical additives (Holt et al., 2018; Liu, 2020). This synergistic integration of microbial ecology and product functionality represents a promising frontier in beverage biotechnology.

Research into the metabolic relationships among microbial groups, such as the interactions between acetic acid bacteria and yeast during beverage maturation, provides critical insights for refining acid balance and developing more complex aroma profiles. During this stage, processes such as diacetyl reabsorption and further transformation of fermentation by-products significantly shape the final sensory quality of beer and wine (De Roos et al., 2018; Spitaels et al., 2014). These interactions are significant in specialty sour and barrel-aged beverages, where subtle biochemical exchanges govern prolonged aging processes, further enriching flavor complexity and acidity modulation. Mastering these microbial dynamics enables the development of refined, differentiated products tailored to niche markets that demand elevated sensory sophistication.

Widespread industrial adoption of these biotechnological advances requires substantial investment in molecular microbiological monitoring tools, including strain tracking and genomic analysis (Marsh et al., 2014; Masneuf-Pomarede et al., 2016). Establishing pilot-scale validation frameworks is imperative for translating innovations derived from spontaneous fermentations (using native microbial consortia) into scalable, reproducible manufacturing protocols. Both scalability and reproducibility ensure rigorous quality control while accelerating the industrial deployment of novel microbial communities (Marsh et al., 2014).

## 5 The importance of native microorganisms in sustainability and biodiversity conservation

Native microorganisms isolated from natural environments associated with craft beer and wine production constitute essential elements of the microbial terroir concept. This microbial terroir profoundly influences the distinctive sensory and biochemical profiles of traditional fermented beverages (Capozzi et al., 2015; Paramithiotis et al., 2024). Leveraging these native microbial communities directly supports sustainability and the conservation of local biodiversity, which faces increasing threats from industrial-scale homogenization and commercial starter culture standardization. The deployment of native microbiota substantially reduces reliance on commercial starter cultures predominantly supplied by multinational corporations, fostering enhanced production autonomy and system resilience (Burini, 2022; Grijalva-Vallejos et al., 2020, 2021; Rivas et al., 2025).

**Figure 5:**
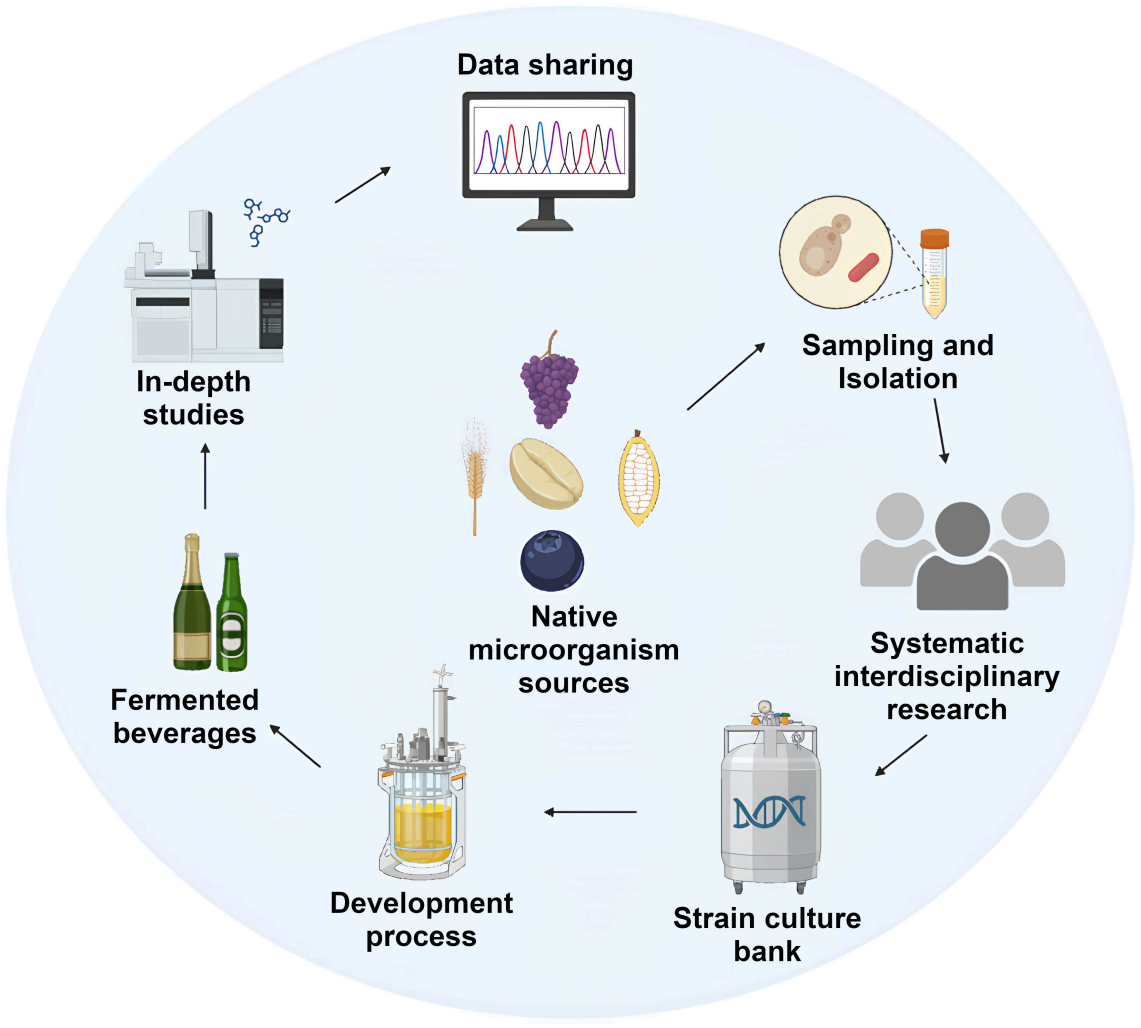
The bioprospecting process: From discovery to application. Created with BioRender.com.

Exploiting native microbes facilitates the preservation and revitalization of traditional and ancestral fermentation methodologies (Bhalla, 2017; Spitaels et al., 2015), thus safeguarding the cultural heritage and regional identity integral to craft production (Burini, 2022; Pirrone et al., 2025). Spontaneous fermentations led by native microorganisms reveal intricate microbial interactions and complex metabolic networks that are frequently overlooked in industrially standardized processes (Grijalva, 2019; Spitaels et al., 2014). Reconnecting with these ancestral practices sparks new scientific insights and opens the door to bioprospecting, pushing fermentation technology toward environmentally sustainable resource-efficient frameworks (Figure 5). Such methodologies reduce consumption of raw materials, energy, and labor, align with circular economy principles and thereby reduce environmental impact.

Beyond their contributions to sustainability and cultural preservation, native microorganisms serve as powerful biological control agents against spoilage and pathogenic microbes that can reduce fermentation stability, product quality, and shelf life. Their metabolisms include the biosynthesis of metabolites, such as organic acids, bacteriocins, and other antimicrobial compounds, that have inhibitory effects on undesirable microbial populations (Berbegal et al., 2017; Pires and Brányik, 2015). This biocontrol functionality significantly reduces or eliminates the need for chemical preservatives, facilitating the production of cleaner-label beverages while enhancing consumer health and safety (Berbegal et al., 2017; Burini, 2022). Native microorganisms contribute to consistent quality outcomes that are vital for both craft and industrial operations, by stabilizing the fermentation environment and preserving product integrity. A study conducted by (Alvarez et al. 2024) reported that *Kluyveromyces marxianus* strains exhibited antagonistic activity against intestinal pathogens (e.g., *Salmonella enterica*), acting as beneficial microbiota, and that *Pichia manshurica* strains demonstrated antagonistic capacity against pathogens in cell models, both species isolated from fermented beverages, such as kefir. *Metschnikowia pulcherrima* has been widely studied in wine for its ability to produce antagonistic compounds and compete with undesirable yeasts during fermentation, and is used in biological control in winemaking (Martín et al., 2024). Among bacteria with antagonistic functions, *Lactiplantibacillus plantarum* has been investigated in fermented beverages for its antimicrobial and functional activity. (Lin et al. 2025) reported that this bacterium produces organic acids and compounds with the potential to inhibit pathogens or undesirable microorganisms during fermentation. Consequently, the ecological role of native microorganisms as biocontrol agents is important at the intersection of microbial ecology, food safety, and sustainable production (Capozzi et al., 2019; Dysvik et al., 2020a).

## 6 Challenges and future perspectives

The use of native microorganisms in the production of fermented beverages presents challenges that must be addressed to optimize and standardize production processes (Alencar and Hikichi, 2019; Burini, 2022). First, the unpredictability of spontaneous fermentations, which are mediated by native microorganisms, is the main challenge to be addressed. These fermentations often exhibit high variability due to fluctuations in microbial composition and environmental conditions (Praveen and Brogi, 2025). The unpredictability complicates the standardization of control parameters and quality specifications necessary to ensure consistent product quality. Furthermore, this variability limits the scalability of processes to industrial levels since reproducibility and product safety can be compromised (Alencar and Hikichi, 2019; Praveen and Brogi, 2025; Qin et al., 2025).

Spontaneous fermentation dynamics represent a complex challenge due to the continuous interactions between microbial groups, particularly the succession and competition between yeasts and LAB documented in Lambic and Sour beers and mirrored in spontaneous wine fermentations (Spitaels et al., 2014; Yoncheva et al., 2024). Although studies of spontaneously fermented beers and wines have begun to map these successions and community shifts, our current understanding of these microbial ecologies remains quite limited, making these interactions difficult to control and standardize. Thus, efforts should be made to maintain quality and the desired characteristics during the upscaling of craft wine or beer production.

Another critical obstacle lies in the genetic diversity and selection of specific strains with beneficial traits for sustained production (Alencar and Hikichi, 2019; Praveen and Brogi, 2025). The identification and selection of suitable strains require advanced molecular biology techniques for species and strain differentiation, combined with multi-omics analyses. While these methods are indispensable to guarantee that selected microorganisms confer desirable attributes, they are costly and time-intensive (Ellis et al., 2022; Pieczonka et al., 2020). Consequently, the rapid adoption of native microorganisms in industrial settings is limited.

The environmental context in which native microorganisms develop significantly influences their isolation success and fermentation performance (Burini, 2022; Fernández, 2018; Grijalva, 2019). These organisms and their fermentation processes depend heavily on specific environmental conditions and the characteristics of raw materials. Even subtle changes in temperature, pH, nutrient availability, or agricultural practices can modify microbial behavior, ultimately affecting quality and sensory attributes of the final products (Fernández, 2018; Grijalva, 2019).

The risk of contamination poses a critical challenge when employing native microorganisms. Certain strains may produce excessive biomass or undesirable compounds known as off-flavors, negatively affecting sensory acceptance. Such risks require rigorous monitoring to prevent undesirable microbial populations that could affect the safety and quality of wine or beer (Burini, 2022; Fernández, 2018).

Co-inoculation or sequential inoculation strategies are effective approaches that use native microorganisms in conjunction with standardized species. These techniques mitigate the inherent risks of spontaneous fermentation by reducing the incidence of low-quality products while simultaneously improving the sensory and functional profiles of fermented beverages (Alencar and Hikichi, 2019; Bader et al., 2019).

Conventional and advanced genetic improvement techniques offer opportunities to optimize specific strains of native microorganisms. For instance, targeted gene suppression can eliminate pathways responsible for off-flavor production, thereby enhancing organoleptic qualities and product stability. These innovations open the way for more controlled, customizable fermentations that balance tradition with technological precision (Martínez et al., 2025).

## 7 Recommendations and concluding remarks

Native yeasts and LAB synergistically contribute to fermentation dynamics, modulating critical parameters such as ethanol yield, aroma profile, acidity, and mouthfeel, ultimately defining product identity and consumer experience. Integrating native microorganisms into craft beer and wine production has emerged as a novel tool for innovation, offering transformative impacts on fermentation quality, sensory complexity, and product authenticity. This approach integrates the preservation of microbial biodiversity with the exploitation of unique metabolic capabilities that drive terroir expression and regional distinctiveness, thereby addressing the classical homogenization prevalent in industrial monoculture fermentations.

Despite their evident benefits, the practical application of native microbial communities isolated from spontaneous fermentations faces several important challenges. Variability, driven by fluctuating community composition and changing environmental conditions, reduces process predictability, standardization, and industrial scalability. Moreover, microbial interactions during fermentation are highly complex and remain only partially understood, making it difficult to anticipate community behavior and fermentation outcomes. Addressing these limitations requires sophisticated molecular and biochemical tools for precise strain-level characterization, selection, and management.

Advances in omics technologies and microbial ecology offer promising alternatives to overcome these challenges. Metagenomic, proteomic, and metabolomic approaches enable comprehensive characterization of native microbial communities and support the targeted selection of specific strains with desirable functional traits, thereby optimizing fermentation performance and product quality. In practical terms, co-inoculation and sequential fermentation strategies that combine selected native microorganisms with well-characterized commercial starter strains provide a pragmatic route to improve fermentation control while preserving the sensory complexity typically associated with spontaneous fermentations.

Recommendations for the craft brewing and winemaking industries include investing in multidisciplinary research that integrates microbial ecology, sensory science, and process engineering to deepen understanding of native microbial ecosystems. Emphasis should be placed on developing scalable protocols that maintain terroir authenticity while achieving industrial consistency and food safety standards. Stakeholders must also endeavor to educate consumers about the value of native microbiomes in enhancing product quality and sustainability, fostering market acceptance of innovation rooted in tradition.

Embracing native microorganisms as both custodians of cultural heritage and drivers of innovation holds unprecedented potential to enrich the sensory, functional, and ecological dimensions of craft beverages. Such integration supports biodiversity conservation and regional economic vitality and aligns with global trends that demand authenticity and sustainability. Continued collaborative efforts between academia and industry are essential to secure the resilience and evolution of local craft brewing and winemaking.
